# Revisiting the Genetics of Hypophosphatasia

**DOI:** 10.1002/jimd.70083

**Published:** 2025-10-05

**Authors:** Priya S. Kishnani, Catherine Rehder, Keiichi Ozono, Jordi Pérez‐López, Guillermo del Angel, William R. Mowrey, Meena Balasubramanian, Wolfgang Högler, Eric T. Rush

**Affiliations:** ^1^ Duke University Medical Center Durham North Carolina USA; ^2^ ISEIKAI International General Hospital Osaka Japan; ^3^ Global Medical Affairs, Alexion, AstraZeneca Rare Disease Boston Massachusetts USA; ^4^ Centre for Genomics Research, Discovery Sciences, Biopharmaceuticals R&D, AstraZeneca Boston Massachusetts USA; ^5^ Bioinformatics and Data Science, Alexion, AstraZeneca Rare Disease Boston Massachusetts USA; ^6^ Division of Clinical Medicine, School of Medicine and Population Health University of Sheffield Sheffield UK; ^7^ Sheffield Clinical Genomics Service Sheffield Children's NHS Foundation Trust Sheffield UK; ^8^ Department of Pediatrics and Adolescent Medicine Johannes Kepler University Linz Linz Austria; ^9^ Department of Metabolism and Systems Science University of Birmingham Birmingham UK; ^10^ Children's Mercy Kansas City Kansas City Missouri USA; ^11^ University of Missouri – Kansas City School of Medicine Kansas City Missouri USA

**Keywords:** alkaline phosphatase, bone mineralization, dominant‐negative effect, genetic counseling, genetic screening, genetic testing, genome sequencing, genotype, phenotype, vitamin B_6_

## Abstract

Hypophosphatasia (HPP) is a rare, inherited monogenic disorder that is typically caused by variants in the tissue‐nonspecific alkaline phosphatase (*ALPL*) gene. Genetic testing for *ALPL* variant(s) to confirm the diagnosis in patients with suspected HPP is a standard practice based on availability. This review attempts to improve the current understanding of the genetics of HPP as it addresses five key related topics: (1) HPP patterns of inheritance and the relationship between HPP genotype and phenotype, (2) how the disease can manifest (including specific genotypes) in heterozygotes, (3) potential reasons why some patients have persistently low alkaline phosphatase activity yet lack an *ALPL* variant, (4) the implications of and resources for variants of uncertain significance (VUS), and (5) recent information on genetic testing in fetuses and newborns. We summarize pertinent information applicable in daily clinical practice, with the objective of preventing missed, delayed, or incorrect HPP diagnoses and improving patient care.

## Graphical abstract

Overview of the genetic concepts in hypophosphatasia reviewed in this manuscript.

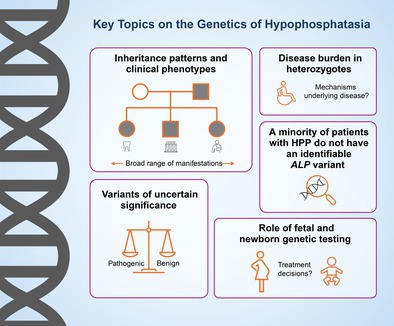

## 1 Introduction

**FIGURE 1:**
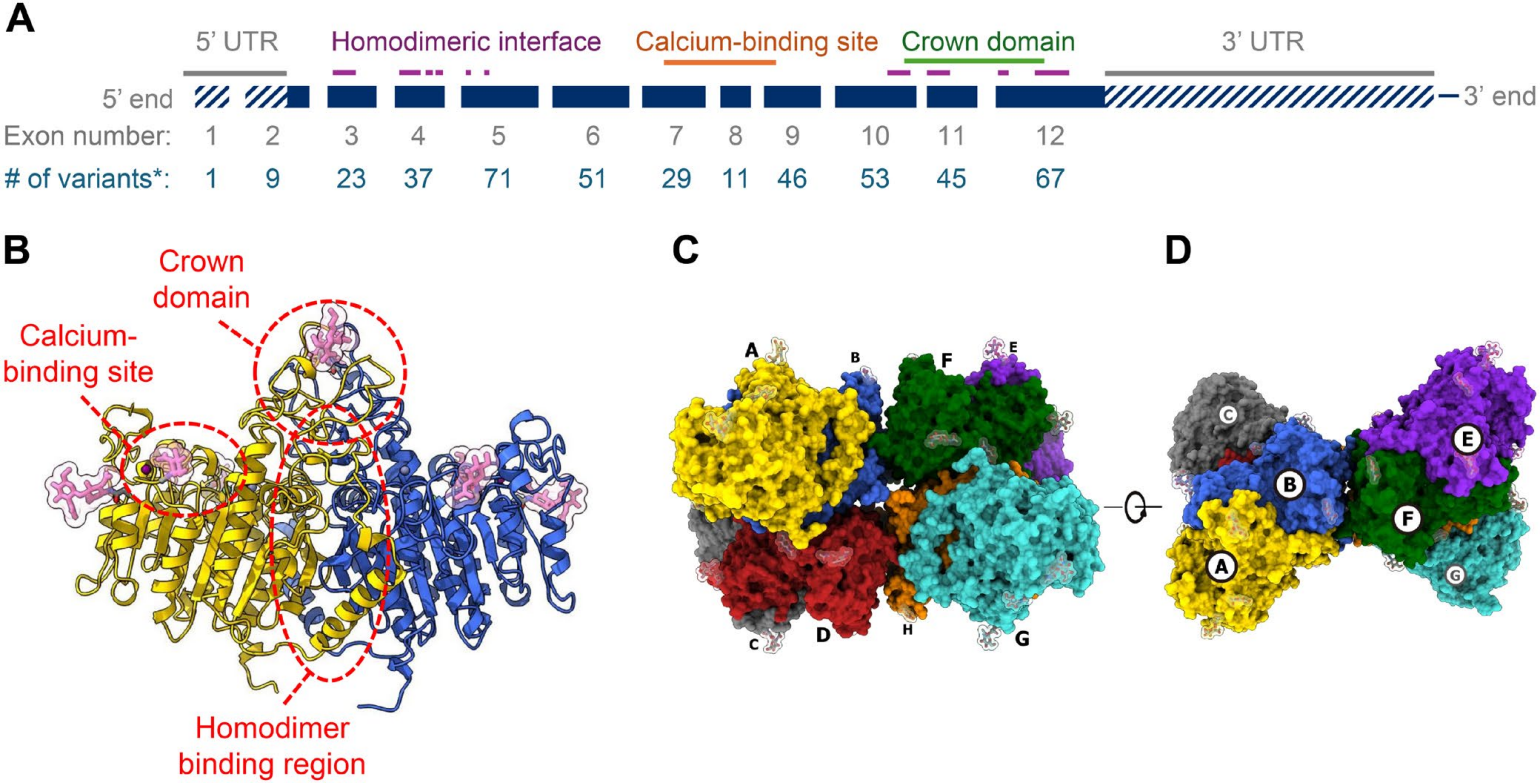
(A) *ALPL* gene map showing exons, introns, and UTRs [1]; (B) ALP dimer structure with sugar moieties shown in pink [2]; (C, D) higher order octamer structure of ALP, shown from two different angles. Protomers are labeled A–H in panels C and D. Panels B–D adapted or reproduced from Yu et al., 2023, under a Creative Commons Attribution 4.0 International License (https://creativecommons.org/licenses/by/4.0/) [2]. ALP, tissue‐nonspecific alkaline phosphatase; UTR, untranslated region. *Number of variants that have been classified in the JKU *ALPL* variant database.

Hypophosphatasia (HPP) is a rare, inherited disease caused by pathogenic variants in tissue‐nonspecific alkaline phosphatase (ALP; gene name: *ALPL* [NM_000478.6]; Figure 1) [3, 4, 5]. The hallmark of HPP is persistently low age‐ and sex‐adjusted serum ALP activity [6, 7]. Deficient ALP activity can lead to extracellular accumulation of substrates including inorganic pyrophosphate (PPi, a potent inhibitor of bone mineralization), pyridoxal 5′‐phosphate (PLP, the active form of vitamin B_6_), and phosphoethanolamine [8, 9, 10]. Certain skeletal and neurologic manifestations of HPP can be partly tied to accumulation of PPi and PLP, respectively [8, 9, 11].

Infants with HPP may present with rickets, vitamin B_6_‐responsive seizures, respiratory failure, muscular hypotonia, hypercalciuria, and nephrocalcinosis, manifestations that can be life‐threatening if left untreated [12, 13, 14]. Children with HPP often experience premature loss of deciduous teeth and have skeletal abnormalities such as rickets, musculoskeletal pain, abnormal gait, and muscle weakness [15]. Adults with HPP frequently report musculoskeletal pain, dental manifestations, fatigue, muscle weakness, and pseudogout [16]. Some patients have a history of recurrent and poorly healing fractures [16].

HPP is diagnosed based on clinical and biochemical findings [7]. Persistently low age‐ and sex‐adjusted serum ALP activity is an obligate criterion for the diagnosis of HPP, although many other factors or conditions can lead to low ALP activity, including intensive care status or severe illness (e.g., cancer) causing low bone turnover, hypoparathyroidism or hypothyroidism, vitamin D intoxication, low magnesium, Celiac disease, and antiresorptive therapies [6, 7, 17, 18, 19]. Elevated plasma PLP and urinary phosphoethanolamine are assessed by ordering vitamin B_6_ and urine amino acids, respectively. These elevated substrates of ALP support a diagnosis of HPP [6]. A scoring system for assessing the likelihood of HPP includes these biochemical features in combination with unambiguous clinical features (e.g., presence of rickets/osteomalacia, early loss of deciduous teeth) [20].

Genetic testing for *ALPL* variants is currently the standard of care for patients with suspected HPP and should be performed whenever possible [7]. Children and adults with low ALP activity and disease manifestations overlapping those of other diagnoses benefit from confirmatory testing to diagnose HPP; this should be done before considering treatment.

Most of the 495 nucleotide and structural *ALPL* variants currently implicated in HPP are missense, with frameshift and other types present in smaller proportions of patients [21, 22]. These variants have been recorded in the Johannes Kepler University *ALPL* gene variant database [20].

Our understanding of the genetics of HPP continues to increase through ongoing research (such as analyses of data from the Global HPP Registry [23]), growing innovation, and availability of next‐generation sequencing technologies. The objective of this review is to provide an overview of the current state of knowledge of HPP genetics with the hope of improving diagnosis and management of HPP. Each topic is first described in a one‐paragraph synopsis, followed by a more detailed background on relevant topics in genetics for clinicians from other disciplines and current evidence in HPP.

## 2 Inheritance Patterns and Genotype–Phenotype Correlations in HPP

### 2.1 Synopsis

HPP is a Mendelian disorder with a mixed pattern of inheritance that is driven by variations in a single gene (*ALPL*). *ALPL* variants can be inherited in an autosomal dominant or autosomal recessive manner, and both inheritance patterns have been reported in the same family. The overall spectrum of HPP ranges from asymptomatic carriers to patients with life‐threatening, early‐onset disease that first manifests before 6 months of age. The heterogeneity of outcomes within families is partly explained by inheritance patterns and, in monoallelic disease, may be further characterized by incomplete penetrance or variable expressivity. There is a limited correlation between genotype and phenotype in HPP. However, phenotypic outcomes are more definitively tied to genotype among infants with early‐onset HPP, who typically have biallelic pathogenic variants [24]. In addition, patients with homozygous c.1559delT or c.1001G>A variants have historically been associated with lethal outcomes, as discussed below.

### 2.2 Genetics Background

Many genetic diseases that follow Mendelian inheritance patterns are monogenic (variants in only one gene) and exhibit one of two main inheritance patterns: autosomal dominant and autosomal recessive [25, 26]. In autosomal dominant inheritance, affected individuals are described as monoallelic or heterozygous, since only one of their two alleles carries a pathogenic variant [25, 27]. In autosomal recessive inheritance, affected individuals may be either homozygotes, with two copies of the same variant, or compound heterozygotes *in trans*, with two different genetic variants on each allele [27]. These patients are described as having biallelic disease since both alleles carry a pathogenic variant [25, 27]. In other rarer cases, an individual may carry a variant that was not inherited from a parent; these variants are de novo [28]. Rarer still, disorders may be inherited via uniparental disomy, in which two variants are inherited from a single parent, resulting in biallelic disease [29, 30].

Mendelian disorders often display nuances that contribute to variable disease presentations, including incomplete penetrance and variable expressivity. Incomplete penetrance refers to a phenomenon in which patients do not display the phenotype that is expected based on their genotype, and variable expressivity refers to the wide variety of clinical manifestations and degrees of severity that can be experienced by patients who have the same genetic variant [31]. As such, the clinical expression of genetic diseases is more challenging than simple dominant or recessive inheritance.

### 2.3 Supporting Evidence in HPP

HPP is a Mendelian disease caused by pathogenic variants in *ALPL* [32]. Similar to other monogenic disorders, clinical features often vary across patients [31]. In HPP, the spectrum of disease includes asymptomatic carriers, individuals with subclinical HPP, and patients with early‐ or late‐onset HPP. These terms describe the presence of genetic, biochemical, and clinical features of disease. Asymptomatic carriers are defined as those who carry an *ALPL* variant but have no biochemical or clinical manifestation of disease. Individuals with subclinical HPP carry an *ALPL* variant and exhibit the biochemical signature of HPP (i.e., persistently low ALP in the absence of other diagnoses or conditions and accumulation of ALP substrates [7, 33]) but have no overt clinical manifestations. Patients with early‐onset HPP first experience clinical manifestations of disease at < 6 months of age, and the disease may be life‐threatening in these patients. Patients with late‐onset HPP have the first onset of disease at ≥ 6 months of age and do not experience life‐threatening manifestations.

Autosomal dominant and autosomal recessive inheritance are both reported in HPP, with some studies reporting both modes of inheritance within the same family [34, 35, 36]. Most recurrent *ALPL* variants are due to founder effects, although there are infrequent case reports of HPP from *de novo* variants [37, 38, 39, 40]. While rare, two cases of HPP inherited via uniparental disomy have also been reported [29, 30]. The vast majority of patients with life‐threatening, early‐onset HPP have biallelic disease, although very rarely patients can have life‐threatening, monoallelic disease, as discussed for c.1559delT below [24, 32, 41]. Dominant inheritance of HPP is rarely apparent in infants; more likely, a second variant may have gone undetected during genetic testing as a result of limitations in sequencing technology, as discussed in Section 3 [24, 32, 42]. Patients with dominant inheritance typically manifest initial symptoms of HPP in childhood or adulthood [24, 32, 42]. Collectively, patients with biallelic disease are typically younger at diagnosis than those with monoallelic disease [24, 43, 44]. While the mode of inheritance in HPP may predict clinical outcomes to a limited extent, a wide range of phenotypic variability is still present among patients, potentially owing to incomplete penetrance and variable expressivity.

The pedigrees of families with HPP often suggest incomplete penetrance. In one instance of a pedigree spanning three generations, the grandmother and father both had a single c.571G>A variant and no clinical features of HPP [45]. ALP activity was low in the grandmother [45]. The proband was a granddaughter who had the same heterozygous c.571G>A variant but presented with short stature and multiple fractures, including one that resulted from minimal trauma [45]. This suggests that the c.571G>A variant is most likely dominant with variable expressivity. This finding was corroborated by an analysis of data in the UK Biobank showing that of 14 heterozygous patients who carried a c.571G>A variant and had repeated ALP measures, only 2 (14%) had persistently low ALP activity [46]. Additional clinical and biochemical features, not available in the UK Biobank, would be required to make an HPP diagnosis in these patients. Thus, the frequency of HPP in c.571G>A heterozygotes is likely lower than the 14% meeting the obligate criterion of persistently low ALP activity.

**FIGURE 2:**
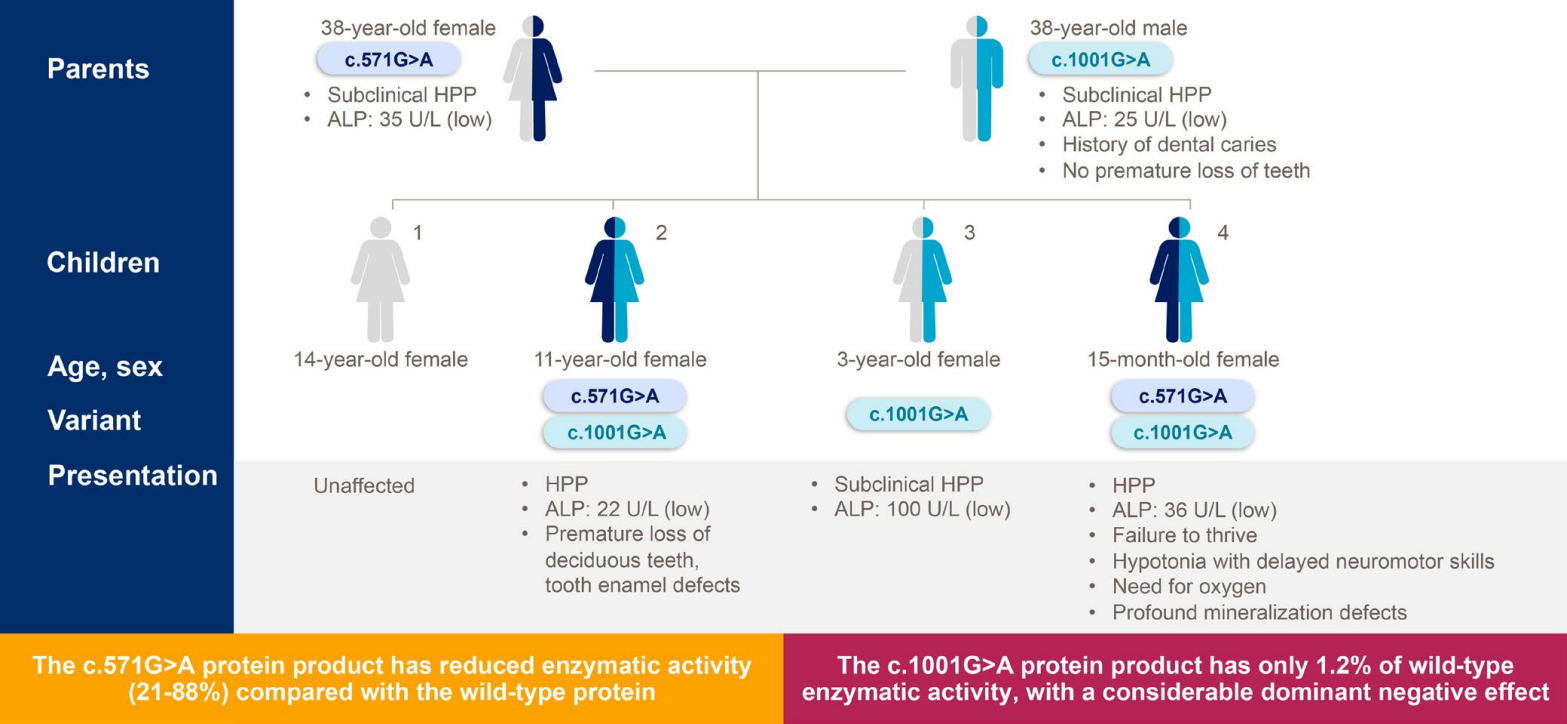
Different phenotypes can occur among family members with the same *ALPL* variant. Figure adapted from Hofmann, et al., 2019 [4, 45, 47].

Pedigrees also demonstrate variable expressivity among family members who carry the same *ALPL* variant(s) (Figure 2) [4, 48, 49]. In one family, two children with identical compound heterozygous c.[571G>A];[1001G>A] variants presented differently from one another: one presented with failure to thrive, recurrent vomiting, and difficulty swallowing, while the second presented with dental manifestation, knock knees, and muscle soreness (Figure 2) [48]. ALP activity was below the reference range in both children [48]. The same c.[571G>A];[1001G>A] genotype has been reported in additional patients, including two siblings diagnosed in the first year of life who presented with failure to thrive and an adult woman with a history of musculoskeletal pain, fractures, and dental problems [48]. Less heterogeneity is observed in patients with autosomal recessive inheritance of variants associated with life‐threatening, early‐onset disease.

Similar variability in disease presentation has been reported among family members who are heterozygous for the same pathogenic variants. For example, a woman with low ALP activity, elevated PLP and urinary PEA, and a c.1231A>G *ALPL* variant had a history of frequent headaches, brain fog, balance problems, dental caries, bone pain, and metatarsal fracture, while her brother, who had the same heterozygous variant, had early loss of primary teeth, bone pain, and low ALP activity [35]. To date, the role of sex hormones in the regulation of *ALPL* gene expression and variable phenotypes is unknown.

Finally, multiple pedigrees have documented absent, subclinical, or late‐onset disease among parents who each carry a single *ALPL* variant, and life‐threatening disease among their children who inherit both variants, suggesting a dosage effect [35]. In one family, the mother was diagnosed with autosomal dominant HPP based on the presence of severe musculoskeletal pain, fatigue, dental problems, and a pathogenic c.331G>A *ALPL* variant [35]. The father had a pathogenic c.1426G>A variant and a history of poor dentition, but no biochemical workup was conducted. Their daughter, who had both variants present, died of HPP‐related respiratory insufficiency at age 31 days. In this family, the mother presented with autosomal dominant inheritance, whereas the father was classified as an asymptomatic carrier. Collectively, inheritance of HPP in this family is consistent with variable expressivity [35].

This culmination of factors collectively results in a limited correlation between genotype and phenotype in HPP [48, 50]. Despite this limitation, some genotypes are more definitively tied to distinct clinical outcomes in patients with HPP, particularly genotypes associated with life‐threatening, early‐onset disease. Homozygous c.[1559delT];[1559delT], which is prevalent in the Japanese general population, is associated with lethal outcomes in infants [51, 52]. Heterozygotes carrying a single c.1559delT variant have a variety of phenotypic outcomes, ranging from life‐threatening to subclinical (defined as having an *ALPL* variant and/or the biochemical phenotype of HPP without an overt clinical phenotype) [41, 53]. It is likely that patients with life‐threatening disease and a single c.1559delT variant harbor a second, undetected variant that contributes to their high disease burden. Compound heterozygotes carrying a c.1559delT variant and a second pathogenic variant experience a range of clinical features, although they are unlikely to have subclinical disease [51, 54, 55]. Homozygosity for c.1001G>A, which is prevalent among Mennonites in North America, is also associated with lethal outcomes [56, 57]. As with patients carrying a c.1559delT variant, patients with compound heterozygous or single heterozygous c.1001G>A variants have variable phenotypes and ages at first presentation [22, 57]. All reported HPP phenotypes associated with different *ALPL* genotypes and variants, including c.1559delT and c.1001G>A, can be viewed in the *ALPL* gene variant database (https://alplmutationdatabase.jku.at/table/) [20].

## 3 Symptom Manifestation and Disease Burden in Heterozygotes With HPP

### 3.1 Synopsis

Up to 83% of patients with late‐onset HPP are heterozygotes [23]. These patients can have a variable range of clinical signs and symptoms that can confer significant disease burden [2, 44]. Among patients with late‐onset HPP, several metrics of disease burden were similar between patients with 1 vs. ≥ 2 *ALPL* variants [44]. The best characterized phenomenon underlying disease manifestation in heterozygotes is the presence of variants that exert dominant‐negative effects (DNEs) [23]. More speculative reasons include the presence of variant(s) in modifier genes (i.e., additional variant(s) in postulated non‐*ALPL* genes that influence ALP expression or function and thus the HPP phenotype) [58], haploinsufficiency (loss of function) [59], the presence of a second *ALPL* variant not identified by current technology, and hormonal or epigenetic regulation of gene expression.

### 3.2 Genetics Background

Functional tissue‐nonspecific ALP protein operates as a dimer and may form a higher order octamer [4, 5]. If an abnormal protein interferes with the activity of the protein encoded by the normal allele, the variant is said to have a DNE [60]. Once both alleles (wild‐type and variant) are transcribed and translated, the abnormal protein monomer interferes with the activity of the wild‐type monomer, reducing enzyme stability or activity to < 50% of the wild‐type heterodimer [4, 60, 61]. The dominant‐negative protein may also cause impaired subcellular localization, including sequestration of the protein in the Golgi such that it cannot translocate to the cell membrane, which limits enzyme activity at the protein's functional site [60, 61]. Impaired enzyme activity may also be due to haploinsufficiency (loss of function), in which the wild‐type allele cannot produce sufficient amounts of protein to maintain normal function [32].

Disease burden may be influenced by the presence of variants outside the coding regions of the gene that causes the disease or by variants in other genes that contribute to disease outcomes. The presence of *cis* variants (i.e., variants present on the same allele) may be inherited in both the coding region of the target gene and the target gene's regulatory sequence [62]. These variants can alter the expression of functional alleles and potentially impact disease penetrance, thereby contributing to variations in symptom manifestation in heterozygotes [62]. Modifier genes are genes that influence disease outcomes despite not being the target gene [47]. Modifier genes may exert their effects through mechanistic overlap with the same biologic process as the target gene or through direct interaction with the protein product of the target gene. The consequence of these interactions is clinical variation among individuals with the same disease‐causing variants in the target gene. Thus, modifier genes are a potential mechanism underlying variable expressivity seen in single‐gene disorders [47].

### 3.3 Supporting Evidence in HPP

A substantial population of patients who have a diagnosis of HPP are heterozygotes, including up to 83% of patients with late‐onset HPP [23, 37]. This percentage is likely biased toward symptomatic patients who seek medical attention rather than asymptomatic carriers, given the population heterozygote carrier frequency of 1:187–1:274 [37, 63]. Among heterozygotes who are clinically diagnosed with late‐onset HPP, over half report pain and dental symptoms, and a third report constitutional/metabolic and skeletal manifestations [44]. Quality of life scores were below the healthy population average among heterozygous adults. Of note, pain, disability, and quality of life scores in this analysis were not significantly different from those of patients with biallelic disease, indicating that manifesting HPP is characterized by high disease burden regardless of variant state in this patient population [44].

Presence of dominant‐negative variants, as confirmed by in vitro functional testing, is one mechanism that explains disease manifestation in some heterozygotes with HPP [32]. Among 608 heterozygotes in the Global HPP Registry, 27% (164 of 608) had a variant that exerts a DNE according to in vitro testing, and 37% (227 of 608) had a variant with no DNE [23]. The remaining 36% (217 of 608) of patients in this analysis had a variant for which DNE testing was not performed. Classification of variants as dominant‐negative in this analysis was based on previous in vitro functional testing, a transfection study using 100% wild‐type, 100% mutant *ALPL* plasmid, and 50:50 co‐transfection of wild‐type and mutant *ALPL* plasmids [4]. Of 155 *ALPL* variants tested, 90 had low residual activity (≤ 25%) and 24 had a DNE, defined in this study as wild‐type/mutant activity < 0.4 [4].

Heterozygotes can have variants that do not exert DNE and still have HPP. For example, 4.4% of heterozygous patients in the Global HPP Registry have a c.571G>A variant, which does not exert a DNE, although they were considered to have a diagnosis of HPP by their clinician [23]. Residual enzyme activity is variably low (~21%–88%) and penetrance is thought to be low for c.571G>A when inherited in the heterozygous state [4, 23, 49]. Some reports have suggested that HPP in heterozygotes without a DNE is likely due to haploinsufficiency, although this is currently speculative and remains to be characterized [37, 59]. Regardless of variant state, individuals with an *ALPL* variant may never develop signs and symptoms of HPP or may do so later in life; that is, people can develop clinical manifestations of HPP in a cumulative, progressive fashion as they age [64].

Little is known about *cis* variants or potential modifier genes in HPP. The c.787T>C variant in exon 7 of *ALPL* is classified as benign, although it may cause subclinical HPP in the homozygous state or contribute to HPP when inherited with a second *ALPL* variant, even when the second variant is also considered benign [65]. One study identified *COL1A2* as a potential modifier gene of HPP among adults with heterozygous *ALPL* variants, although this finding remains speculative [58]. An exome‐wide association study of data in the UK Biobank identified several genes associated with changes in ALP activity, such as *GPLD1*, *IFITM5*, *APOB*, *PCK1*, and *HSPG2*, which were associated with decreased ALP activity, and *ABCB11*, *ASGR1*, *EDEM1*, *AKAP9*, *SLC39A5*, and *B4GALNT3*, which were associated with increased ALP activity [46]. Other genes, including *GPLD1*, *ABO*, and *REEP3*, have been identified through genome‐wide association studies as modifiers of serum ALP activity and are also of interest for their potential role in modulating the HPP phenotype [66, 67, 68, 69]. Protein products of the genes *ANKH*, *ENPP1*, *PANX1*, and *PHOSPHO1* are potentially of interest in HPP since they regulate the availability of ALP substrates, although these have not been investigated in HPP [70]. ALP activity may also be regulated by changes in hormones, including sex hormones [71], although this remains to be systematically evaluated in patients with HPP. Finally, while speculative, it is possible that heterozygotes with HPP have a second *ALPL* variant that was not detected by genetic sequencing. This is sometimes suspected in heterozygous infants with early‐onset, life‐threatening disease [24].

## 4 Identification of *ALPL* Variants in Patients With HPP

### 4.1 Synopsis

The vast majority of patients with HPP have at least one detectable *ALPL* variant, although occasionally this is difficult to prove with current technology that is applied in clinical practice and the consequent lack of available data. One estimate predicts that approximately 95% of patients are expected to have at least one detectable *ALPL* variant, although the true proportion may be higher [23]. It is unclear whether this reflects false negative test results related to sequencing techniques among patients who in fact have structural variants, insertions, deletions, or cryptic *ALPL* variants deep in intronic, promoter, or other regulatory regions, or whether alternative genes are implicated in HPP manifestations. For that reason, while detection of an *ALPL* variant can help confirm an HPP diagnosis, patients without a detected variant can still be diagnosed with HPP if clinical and biochemical signs are typical and other causes for low ALP activity have been excluded [7]. Investigation of any genes beyond *ALPL* that cause or modify the HPP phenotype is an ongoing research objective.

### 4.2 Genetics Background

When a diagnosis of HPP is suspected, standard practice is to perform sequencing of the coding regions (exons) and intron/exon borders of *ALPL* [32, 72]. Historically, because most sequencing assays did not cover introns and regulatory regions, some variants were missed. For example, sequencing approaches often failed to detect large heterozygous deletions (including whole gene deletions), whole gene duplications, deep intronic variants, and variants in regulatory regions (such as promoters) or untranslated regions [32, 73, 74, 75]. Advancements in next‐generation sequencing are helping address these challenges.

If initial sequencing methods do not detect an *ALPL* variant, alternative strategies may be applied. Genome sequencing (previously called “whole genome sequencing”) analyzes both coding and noncoding regions of DNA and thus provides greater coverage than exome sequencing [76]. While genome sequencing can detect nonexonic variants, some of these variants can be challenging to functionally test and may complicate interpretation of sequencing results. Long‐read sequencing can be used for genome or exome sequencing, and can also detect larger genomic changes not easily detectable by more common short‐read sequencing methodologies [77]. Because alterations in modifier genes can affect *ALPL* expression, RNA‐seq could help identify patients with decreased ALP activity in the absence of an *ALPL* variant.

### 4.3 Supporting Evidence in HPP

Sequencing techniques allow detection of *ALPL* variants in approximately 95% of patients with HPP; however, patients with unambiguous, persistent signs may be diagnosed with HPP even if sequencing fails to identify a variant [23, 78]. Sequencing technologies have evolved considerably over the past several years, and use of the more modern sequencing methods can sometimes reveal variants in patients who first appear to test negative for a variant. For example, multiplex ligation‐dependent probe amplification and deep analysis of branch point sequences have both identified variants that were originally missed by Sanger sequencing in patients with confirmed or suspected HPP [79, 80].

While emerging technology can sometimes detect missed variants, these techniques may still fail to detect variants in some patients. In an analysis of 16 patients who were diagnosed with HPP based on clinical and biochemical manifestations and were originally found to be negative for variants that were either pathogenic, likely pathogenic, or of uncertain significance, none were found to have an *ALPL* variant through genome sequencing [78]. It is unclear if these patients carried *ALPL* variants that remained undetected with genome sequencing or if they carried variants in other genes that may modify the HPP phenotype, as discussed in Section 2.

## 5 Variants of Uncertain Significance (VUS) and Their Role in the Diagnosis of HPP

### 5.1 Synopsis

A VUS is a variant that has not yet been definitively associated with disease or definitively determined not to be associated with disease. Patients who present with clinical and biochemical symptoms consistent with HPP can still be diagnosed with HPP despite carrying a heterozygous *ALPL* VUS. A VUS may be reclassified if substantial additional evidence for a pathogenic or benign classification is generated, or in cases with insufficient evidence, the variant may remain a VUS. The international *ALPL* Gene Variant Consortium comprises HPP expert clinicians, geneticists, genetic counselors, basic scientists, and biocurators who work to reclassify VUS submitted through their submission portal (https://alplmutationdatabase.jku.at/portal/).

### 5.2 Genetics Background

The American College of Medical Genetics and Genomics (ACMG) has five classifications of variants: pathogenic, likely pathogenic, VUS, likely benign, and benign [81]. The terms “likely pathogenic” and “likely benign” conservatively refer to 90% certainty that the variant is disease‐causing or benign, respectively [81]. Variants are classified as VUS when the criteria for the other four classifications are not met or when the criteria for pathogenic or benign are contradictory [81].

Given the conservative nature of this classification system, periodic testing and potential variant reclassification are important for providing clear interpretation of genetic variants. *ALPL* variants, including VUS, are continuously cataloged in the Johannes Kepler University *ALPL* Gene Variant Database (https://alplmutationdatabase.jku.at/) [20]. Through this freely accessible database, healthcare professionals and researchers can search for specific variants to determine their current classifications and access links to cases reporting these variants. Individual clinicians, geneticists, genetic counselors, and researchers may also submit novel variants and VUS to the project team through a submission portal (https://alplmutationdatabase.jku.at/portal/). VUS in the database are currently being assessed and potentially reclassified via the Global *ALPL* Gene Variant Classification project based on criteria from the ACMG and the Association for Molecular Pathology (AMP) [20, 81]. Classification is a multistep process that includes assessing the clinical phenotype suggestive of HPP, searching available literature, evaluating genetic evidence, performing functional testing, conducting a full variant assessment according to modified ACMG/AMP specifications, and completing a consortium review and variant reclassification. Newly classified variants are updated in the *ALPL* gene variant database and are submitted to ClinVar [20].

### 5.3 Supporting Evidence in HPP

**FIGURE 3:**
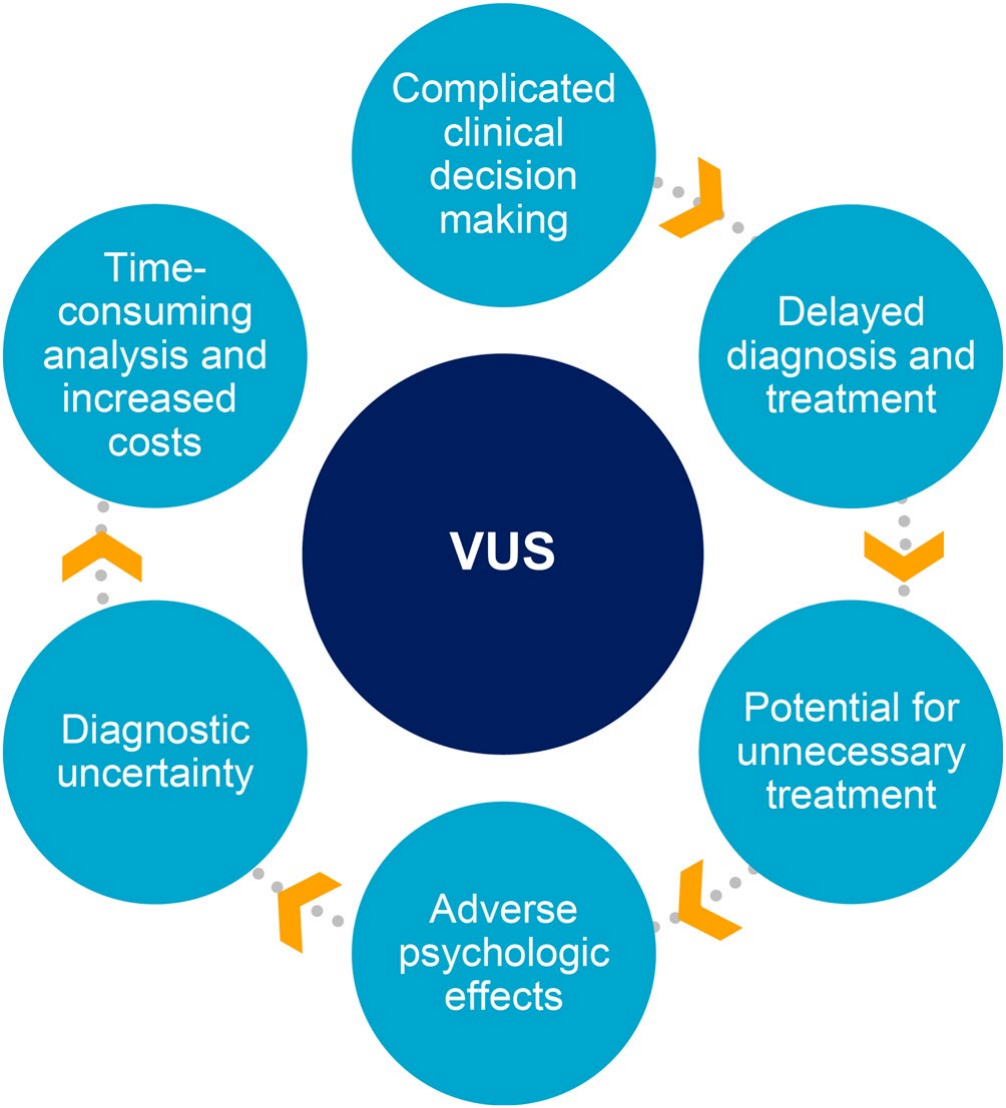
Challenges associated with VUS [82, 83]. VUS, variant of uncertain significance.

Among patients enrolled in the Global HPP Registry, 6.3% of patients have at least one VUS and 4.4% of patients exclusively have a VUS [23]. VUS can create uncertainty about the diagnosis of HPP, potentially leading to delays in appropriate treatment (Figure 3) [20, 84, 85]. HPP is likely in an individual who presents with the biochemical signature of HPP and typical, unambiguous clinical signs and symptoms of the disease, even if they carry a VUS instead of a pathogenic or likely pathogenic variant [7, 20]. Notably, detection of a pathogenic or likely pathogenic *ALPL* variant is not required for diagnosis of HPP [7]. While reclassification of VUS as pathogenic or benign can occur at any time, reports of this in the current literature for HPP are sparse [86, 87]. A recent analysis reclassified 3 VUS (c.69_74del, c.875C>T, and c.1135C>A) as either pathogenic or likely pathogenic among patients who were suspected to have HPP [87]. Importantly, an effort is ongoing through the Global *ALPL* Gene Variant Classification project to examine and reclassify *ALPL* VUS [33]. As of 2025, 100 VUS have been examined for potential reclassification [33]. To date, 73 of these VUS were reclassified as pathogenic or likely pathogenic, 4 were reclassified as benign or likely benign, and 23 remained VUS [33]. Of the 100 variants, 82 were missense, 7 were frameshift, 4 were in‐frame deletions, 2 were intronic variants, 2 were synonymous, 2 were nonsense, and 1 was a splice site variant [33].

## 6 Fetal and Newborn Testing for Familial *ALPL* Variants and the Role of Testing in Treatment Decisions

### 6.1 Synopsis

It is increasingly common for people to obtain prenatal genetic testing to identify an *ALPL* variant(s). Preconception or prenatal genetic testing may be performed based on family history for HPP or upon discovery of skeletal abnormalities on ultrasound. Typically, prenatal genetic testing is performed to better understand risk of disease, as both autosomal dominant and autosomal recessive diseases have implications for disease burden and recurrence risk. Careful counseling of mothers for cases identified in utero via sonogram or genetic testing is required, as it can be difficult to distinguish between developing fetuses likely to be born with life‐threatening vs. nonlife‐threatening HPP.

### 6.2 Genetics Background

Prenatal genetic testing may include single‐gene or multigene sequencing, with family history and clinical signs in utero guiding testing decisions. Known familial variants are typically assessed in utero through targeted sequencing, whereas comprehensive gene panels (e.g., skeletal dysplasia panels) are a more efficient choice when signs are ambiguous on ultrasound and/or when there is no family history of HPP, as discussed in the Background portion of Section 3 [28]. Such testing can be useful for identifying a number of skeletal disorders that are part of the differential diagnosis for HPP, including osteogenesis imperfecta, X‐linked hypophosphatemia, and others [88, 89]. Trio testing, which describes genetic testing of the fetus and both parents, is an effective and useful tool that can increase diagnostic rates and inform clinical decision‐making [90]. Since genetic testing is performed on both parents, this approach also provides valuable insights on genetic phasing in the fetus.

### 6.3 Supporting Evidence in HPP

Detecting HPP in a developing fetus is usually accomplished through a combination of ultrasound and genetic testing. However, sonography is not often definitive to determine if an infant will be born with life‐threatening HPP [91, 92]. First, the clinical signs of impaired mineralization that are detected with ultrasound, including short or bowed long bones, deficient bone ossification, lung hypoplasia, and small, beaded ribs, are ambiguous findings that may point to HPP or to other disorders such as osteogenesis imperfecta [93]. Second, sonography typically cannot distinguish between life‐threatening and nonlife‐threatening HPP [91]. In addition, signs of HPP can improve or stabilize in the third trimester or after birth, complicating early diagnosis based on ultrasound [94, 95, 96]. While these features alone may be clinically ambiguous, elaboration of these findings with family mapping and genetic testing can aid in diagnosis.

Genetic testing can help identify HPP in utero and should be performed if HPP is suspected based on ultrasound findings or positive family history [91, 93]. Life‐threatening HPP is typically inherited in an autosomal recessive manner (i.e., biallelic disease) [91, 95]. As such, detection of multiple *ALPL* variants may shed light on phenotypic outcomes after birth, especially if the fetus has a well‐characterized genotype, such as c.[1559delT];[1559delT] or c.[1001G>A];[1001G>A], which more definitively predict life‐threatening outcomes [51, 57]. It is, however, noteworthy that biallelic disease is also reported in patients with nonlife‐threatening HPP, and the presence of two variants cannot always predict life‐threatening disease, as discussed in Section 1. Obtaining information on ALP activity in the mother (before pregnancy) and father may be a useful strategy for predicting outcomes in the fetus. Prognosis of the developing fetus may also be more predictable if a previous pregnancy or sibling showed the same genetic variant(s), although children with genotypes identical to those of their family members may still have variable phenotypes. Further work is needed to identify additional genotypes, beyond c.[1559delT];[1559delT] and c.[1001G>A];[1001G>A], that may predict a life‐threatening outcome.

Newborn screening tests do not test for HPP and are not currently recommended by the U.S. Department of Health and Human Services as part of the Recommended Uniform Screening Panel. As with pediatric and adult patients, newborns are tested for *ALPL* variants on the basis of clinical manifestations and/or family history. The newborn assessment landscape is likely to change in the near future. Ongoing studies, including the Generation Study in England and a large newborn screening study in China, are being undertaken as public health initiatives to use genetic screening to detect a wide range of conditions in newborns, including HPP [97, 98].

## 7 Conclusions and Future Directions

HPP is characterized by high clinical heterogeneity, even in patients with the same *ALPL* variant(s), suggesting incomplete penetrance and variable expressivity. Heterozygotes with a single *ALPL* variant may be asymptomatic carriers with no biochemical signature or may have subclinical HPP with the biochemical signature of disease but no overt clinical manifestations. Alternatively, heterozygotes may be diagnosed with HPP based on clinical features and can experience appreciable disease burden that may accumulate over the course of their lives. While not all patients have an identifiable *ALPL* variant and some may present with a VUS, this does not preclude diagnosis of HPP, provided that they present with the biochemical signature of HPP and unambiguous clinical manifestations of the disease. Tailored genetic counseling should be given to individuals with suspected HPP, as well as family members of those with suspected or confirmed diagnoses.

HPP is a rare disease and, as such, information is limited. Further research involving large genetic databases, including UK Biobank, All of Us, and BioVU, is needed to help resolve current gaps in the literature on HPP genetics, such as the presence and effects of modifier genes [99, 100, 101]. Investigation of epigenetic factors that may influence the HPP phenotype is also warranted. Such research may help identify sources of variable expressivity in HPP disease manifestations and increase the predictability of clinical outcomes from genotype data. Several genomic screening studies are in progress to assess for HPP and other diseases among newborns, including GUARDIAN (Genomic Uniform‐screening Against Rare Disease in All Newborns), BabySeq, and BeginNGS [1, 82, 102]. These systems may change the genetic testing landscape and have a profound influence on the early detection of inherited metabolic disorders. In a pilot clinical trial among 120 infants in a neonatal intensive care unit, BeginNGS screening had a true positive rate of 4.2% with 83% sensitivity (5 of 6 patients) for all disorders assessed [83]. Patient management was anticipated to change based on findings in each of the 5 patients with true positive results, highlighting the benefit of BeginNGS among infants with genetic disorders treated in the neonatal intensive care unit [83]. Finally, a substantial number of VUS are continuously being reclassified, and new genotypes and associated phenotypes are continuously added to the *ALPL* Gene Variant Database [33], which increases diagnostic certainty, increases information on the phenotypic spectrum, and assists in counseling.

## Author Contributions

Conceptualization: Meena Balasubramanian and Catherine Rehder. Data curation and formal analysis: Meena Balasubramanian. Validation: Wolfgang Högler, Meena Balasubramanian, and Eric T. Rush. Writing – original draft: All authors. Writing – review and editing: All authors.

## Conflicts of Interest

Priya S. Kishnani consults for and has received research funding and honoraria from Alexion, AstraZeneca Rare Disease. Catherine Rehder has received salary support from the Johannes Kepler University Linz agreement. Keiichi Ozono has received research funding and honoraria, and support for meetings and/or travel from Alexion, AstraZeneca Rare Disease. Jordi Pérez‐López and William R. Mowrey are employees of Alexion, AstraZeneca Rare Disease and may own stock/options in AstraZeneca. Guillermo del Angel is an employee of AstraZeneca and may own stock/options in AstraZeneca. Meena Balasubramanian has received research grants, consulting fees, payment or honoraria, and support for meetings and/or travel from Alexion, AstraZeneca Rare Disease. Wolfgang Högler consults for and has received research funding and honoraria from Alexion, AstraZeneca Rare Disease, and BioMarin. He has received support for meetings and/or travel from BioMarin, Novo Nordisk, Sandoz, and Alexion, AstraZeneca Rare Disease. Eric T. Rush has received research funding to his institution from Alexion AstraZeneca Rare Disease and Ultragenyx. He has received consulting fees and payment or honoraria from Alexion, AstraZeneca Rare Disease, Ultragenyx, Inozyme, Ipsen, and Kyowa Kirin. He has received support for meetings and/or travel from Alexion, AstraZeneca Rare Disease and Kyowa Kirin and participates on a data safety monitoring or advisory board for Inozyme.

## Supporting information


**Supplementary Data:** jimd70090‐sup‐0001‐Data.docx.

## Data Availability

Alexion, AstraZeneca Rare Disease will consider requests for disclosure of clinical study participant‐level data provided that participant privacy is assured through methods like data de‐identification, pseudonymization, or anonymization (as required by applicable law), and if such disclosure was included in the relevant study informed consent form or similar documentation. Qualified academic investigators may request participant‐level clinical data and supporting documents (statistical analysis plan and protocol) pertaining to Alexion‐sponsored studies. Further details regarding data availability and instructions for requesting information are available in the Alexion Clinical Trials Disclosure and Transparency Policy at https://www.alexionclinicaltrialtransparency.com/data‐requests/.
