# Supplementary material for: Revisiting the Genetics of Hypophosphatasia
Source: J Inherit Metab Dis. 2025 Oct 5;48(6):e70083. doi: 10.1002/jimd.70083 (PMC12497681; doi:10.1002/jimd.70083)
Supplement: Supplementary file 1 — Supplementary Data: jimd70090‐sup‐0001‐Data.docx. [file JIMD-48-0-s001.docx]

**PLAIN LANGUAGE SUMMARY**

Hypophosphatasia (HPP) is a rare disease caused by low alkaline phosphatase activity due to changes in the *ALPL* gene—these changes are also known as “variants,” and so far, over 480 have been found.

To help physicians better understand HPP genetics and improve early diagnosis of the disease, we discuss five pressing topics on HPP genetics: (1) how HPP is inherited, (2) the relationship between genetic variants in *ALPL* and HPP symptoms, (3) why some patients do not have *ALPL* gene variants, (4) resources for uncertain genetic variants in *ALPL*, and (5) information on genetic testing for newborns.

Providing evidence-based discussion on these important topics will aid clinicians in diagnosing HPP more accurately and improve patient care and outcomes.
